# Ouabain‐induced hypertension in rats: Mechanisms, variability and translational implications

**DOI:** 10.1113/EP092956

**Published:** 2025-08-06

**Authors:** Priscilla Rodrigues O. Feijó, Luis Eduardo M. Quintas

**Affiliations:** ^1^ Laboratório de Farmacologia Bioquímica e Molecular, Instituto de Ciências Biomédicas Universidade Federal do Rio de Janeiro Rio de Janeiro Brazil

**Keywords:** cardiotonic steroids, hypertensive rat model, hypertensive rat model

## Abstract

Ouabain‐induced hypertension is a multifactorial and condition‐dependent phenomenon involving coordinated actions across vascular, renal and central nervous system pathways. At the vascular level, ouabain inhibits Na⁺/K⁺‐ATPase, particularly the α2‐isoform, leading to elevated intracellular Ca^2^⁺, enhanced vasoconstriction and structural remodelling of resistance arteries. These effects are exacerbated by oxidative stress, inflammation, and altered expression of Ca^2^⁺‐mobilizing proteins such as NCX1 and TRPC channels. In the kidney, ouabain disrupts Na^+^ handling, especially in the proximal tubule, suppresses natriuretic pathways like the D1 dopamine receptor, and promotes volume expansion through renal and sympathetic mechanisms. Centrally, ouabain acts on sodium‐sensitive brain regions, including the median preoptic nucleus, rostral ventrolateral medulla and paraventricular nucleus, where it increases sympathetic outflow and impairs baroreflex control. These effects are potentiated by local interactions with brain‐derived angiotensin II and cerebrospinal Na⁺, independent of peripheral ouabain levels. However, the hypertensive response is not universal and may vary by strain, salt status, genetic background and experimental conditions. These insights carry important translational implications. Elevated levels of endogenous ouabain (EO) have been identified in patients with salt‐sensitive, low‐renin or neurogenic hypertension. Therapeutic strategies targeting ouabain‐sensitive pathways include isoform‐selective Na⁺/K⁺‐ATPase modulators, NCX or TRPC inhibitors, and agents acting on the central renin–angiotensin system. EO‐neutralizing therapies such as digoxin antibodies may also hold clinical promise. Personalized medicine approaches incorporating EO sensitivity markers and genotype‐specific models may advance the management of resistant hypertension and deepen our understanding of ouabain's dual role as both physiological modulator and pathological trigger.

## Abstract (highlights)

- **What is the topic of this review?**
  This review examines the exogenous ouabain‐induced hypertensive rat model, emphasizing its use in studying the mechanisms of hypertension and related cardiovascular and renal alterations.
- **What advances does it highlight?**
  It highlights how chronic ouabain infusion induces hypertension through Na⁺/K⁺‐ATPase signalling, vascular remodelling and neurohumoral activation. The model has advanced understanding of salt sensitivity, arterial stiffness and potential therapeutic targets in hypertension research.

## 1 INTRODUCTION

Cardiotonic steroids (CTS) extracted from plants have been used in traditional medicine for several purposes for centuries recapitulating the ancient Egyptians, Romans and Syrians. In the case of digitalis, references to use by the Saxons, Irish and Welsh have been described (Jacobs, 1936). Leonhart Fuchs, in his *De historia stirpium commentarii insignes* (1542) classified the plant botanically and named it *Digitalis* (Rahimtoola, 1975; Withering, 1785), also suggesting diseases, including hydropsy, for which it could be administered as decoctions or infusions (Jacobs, 1936). The English physician William Withering, author of the seminal work *An Account of the Foxglove and Some of its Medical Uses*, published in 1785, used these compounds for the treatment of 163 cases of hydropsy (Jacobs, 1936; Kreis, 2017; Withering, 1785). Later, clinical evidence supported the recommendation of the use of digitalis for some medical conditions, such as ascites and anasarca (M'donald, 1847; Rahimtoola, 1975), and it was believed to be an effective diuretic (Whayne, 2018). In fact, John Ferriar (1799) is considered the first to attribute digitalis to a primary action in the heart, followed by Friedrich Kreysig (1814), but experimental demonstration was conducted by Cattell and Gold (1938).

Several years later, the active pumping transport of Na^+^ and K^+^ was shown to be inhibited by strophantin, a CTS also known as ouabain (Schatzmann, 1953). Na^+^/K^+^‐ATPase (NKA), the biochemical counterpart of the physiological Na^+^/K^+^ pump, was discovered through studies in crab nerves (Skou, 1957) and in 1997 Jens Christian Skou was awarded the Nobel Prize in Chemistry for the discovery of this ion transporter enzyme (Clausen & Persson, 1998). NKA is a transporter that maintains the gradient of Na^+^ and K^+^ ions across the plasma membrane through ATP hydrolysis (Blanco & Mercer, 1998; Leite et al., 2022). It is a P‐type ATPase, a family of transporters/enzymes that interconvert between two different conformations, denoted by E1 and E2. P‐type ATPases contain an aspartyl phosphorylation site and binding sites for the transported ligands, and they catalyse ion transport. Through this mechanism, NKA is essential for several physiological functions such as regulation of cellular osmolarity, maintenance of the resting membrane potential, and excitability of muscle and nerve cells. In the kidney, NKA participates in Na^+^ and water reabsorption and is important for homeostasis of body fluids and electrolytes (Blanco & Mercer, 1998; Geering, 1997; Kinoshita et al., 2016).

NKA is a heteromeric protein composed of three subunits, α, β and γ (FXYD2), and each of them has several isoforms that vary according to the tissue and species (Blanco & Mercer, 1998; Kinoshita et al., 2016). However, the α1β1 dimer shows ubiquitous expression, and the α subunit has the binding site for ouabain and other CTS class compounds (Keenan et al., 2005; Morth et al., 2007; see structure in Leite et al., 2022). Upon binding selectively and reversibly to NKA, a CTS inhibits the activity of the enzyme, which increases the intracellular concentration of Na^+^ due to its reduced extrusion, with consequently a secondary increase in the intracellular concentration of free Ca^2+^, because of a decreased/reverse mode activity of the colocalized Na^+^/Ca^2+^ exchanger (NCX) (Blaustein, 2013). This Ca^2+^ is uptaken by Ca^2+^‐ATPases from the sarcoplasmic reticulum (SERCA), improving Ca^2+^ mobilization. Thus, the greater availability of Ca^2+^ to interact with contractile proteins results in enhanced muscle contraction, known as the cardiac positive inotropic effect (Akera & Brody, 1978).

**FIGURE 1:**
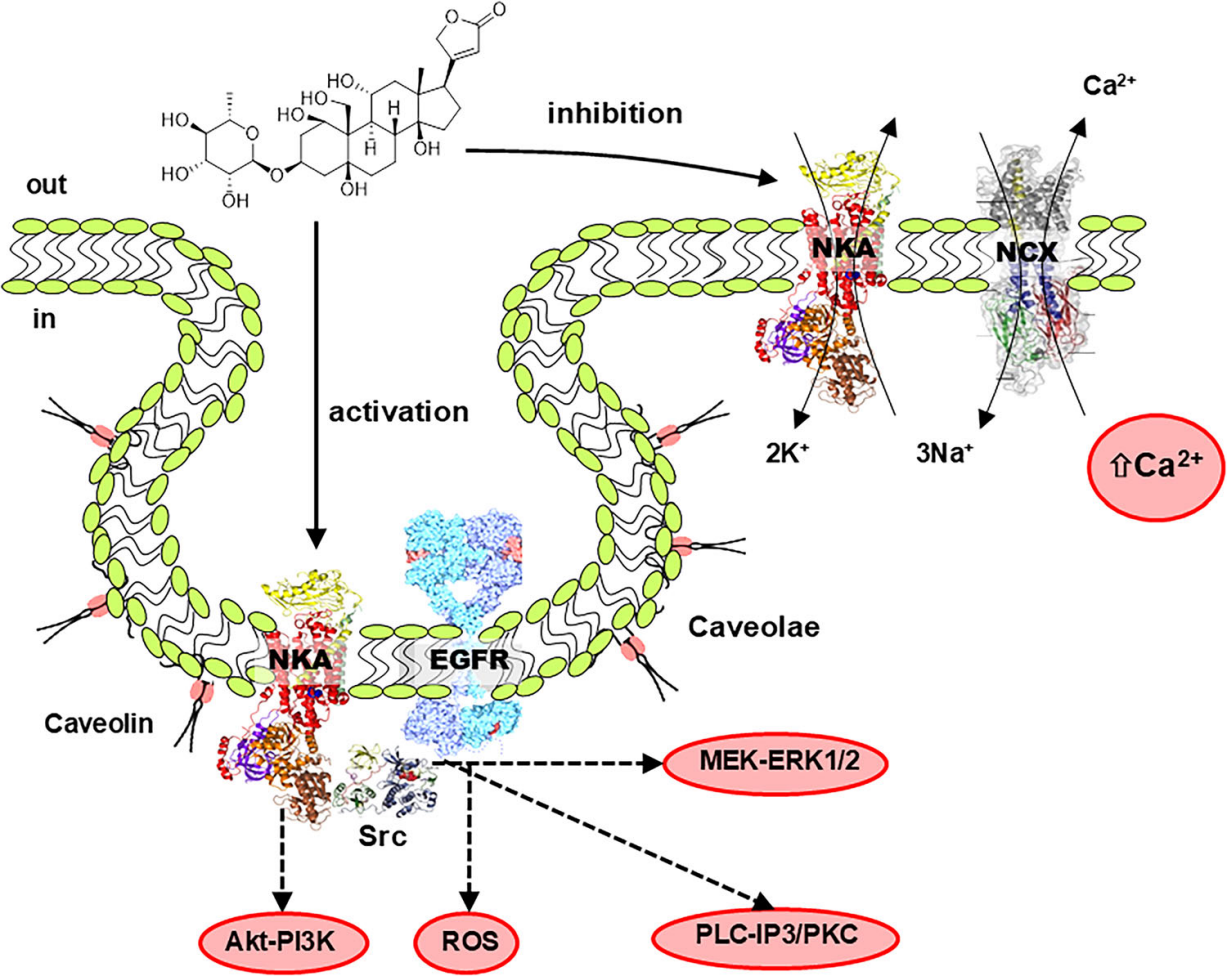
Dual mechanism of action of cardiotonic steroids (ouabain) on Na^+^/K^+^‐ATPase (NKA). The classical mode of ouabain action is the inhibition of NKA in the bulk plasma membrane, which leads to enhanced intracellular Ca^2+^ concentrations by impairment of Na^+^/Ca^2+^‐exchanger (NCX) function. A second mechanism is the triggering of a caveolar NKA‐mediated signalling through Src activation and transactivation of the epidermal growth factor receptor (EGFR), which may stimulate several pathways like mitogen‐activated protein kinase kinase–extracellular signal‐related kinases 1/2 (MEK–ERK1/2), phospholipase C (PLC–inositol trisphosphate (IP3)/protein kinase C (PKC)), and reactive oxygen species (ROS). The Akt–phosphoinositide 3‐kinase (PI3K) pathway was shown to be independent of Src.

In addition to the classic NKA inhibition mechanism, it is currently known that when a CTS binds to the transporter it can also trigger cellular signalling pathways, turning NKA into a signal transducer through protein–protein interactions, as first reported in the seminal work of Xie and Askari (2002). The interaction of a CTS and NKA activates Src tyrosine kinase, in a NKA–Src complex, and the active Src transactivates other proteins such as the epidermal growth factor receptor as well as other serine/threonine kinases, lipid kinases and lipases (Cui & Xie, 2017), giving rise to possible functional selectivity (Amaral et al., 2018). Figure 1 shows the CTS (specifically ouabain) mechanistic duality.

### 1.1 Cardiotonic steroids and ouabain

CTSs have a common structure that includes a *cis–trans–cis* fused steroidal core, which adopts a U‐shaped conformation with a convex β surface, a C14 hydroxyl group (OH14β), and a lactone ring in a β conformation at the C17 position (Agrawal et al., 2012). These compounds are classified into two groups: cardenolides and bufadienolides. The structural difference lies in the heterocyclic unit linked at position 17: 5*H*‐furan‐2‐one‐4‐yl is replaced by pyran‐2‐one‐5‐yl. They may or may not have a C3‐linked glycosidic moiety (Michalak et al., 2017). Our review will focus on the cardenolide ouabain, a hydrophilic CTS, which presents, in addition to the common structure, a rhamnose at C13 (Agrawal et al., 2012; Michalak et al., 2017), and, through those mechanisms of action, participates in several cellular regulatory processes in which the cardiovascular system is prominent (Schöner & Scheiner‐Bobis, 2007). Also, it has been identified in mammals (Hamlyn et al., 1991).

Originally, ouabain was identified in Apocynaceae family plants in the 19th century, from the bark and roots of the African shrub species *Acokanthera ouabaïo* (or *schimperi*, *wabajo…*) and from the seeds of the plant *Strophanthus gratus* (*kombé*, *hispidus*…) (Ouabain, 1932; Blaustein & Hamlyn, 2024; Michalak et al., 2017). Like digitalis preparations, it was used to treat asthma (Ouabaïne, 1891) and whooping cough (Gemmell, 1890) and was commercialized as Ouabaïne Arnaud by the Laboratoire Nativelle (Paris, France) as pills and intramuscular ampoules for cardiovascular conditions (Fürstenwerth, 2010; Hasenfratz, 1932). Indeed, ouabain and digitalis had barely similar clinical indications (Fürstenwerth, 2010, 2019). Endogenous CTSs have been considered a possibility for decades in mammals (Blaustein & Hamlyn, 2020; Kinoshita et al., 2022). However, it was Hamlyn et al. (1991) who identified and characterized for the first time a compound biologically, structurally and immunologically indistinguishable from ouabain in human plasma, suggesting the endogenous production of this CTS (Blaustein, 2018; Hamlyn et al., 1982, 1987; Pavlovic, 2020). This was confirmed after mass spectrometry analysis and high‐performance liquid chromatography, and it was concluded that the endogenous factor was ouabain or a closely related isomer (Hamlyn et al., 1991, 2014; Murrell et al., 2005).

Although the physiological and pathological roles of endogenous ouabain (EO) are still largely unknown, it has been related to long‐term changes in Na^+^ balance and cardiovascular structure and function, being considered a new steroid hormone in mammals, with adrenals as its main source (Doris et al., 1996; Laredo et al., 1995), but it is also produced in the hypothalamus (Kawamura et al., 1999; Blaustein & Hamlyn, 2020). Synthesis and release of EO occur in response to volume expansion, angiotensin II (Ang II) and stimulation by adrenocorticotropic hormone (Shah et al., 1999). In the adrenal gland, EO seems to be the product of a steroidogenic pathway (Simonini et al., 2018), resulting from the cleavage of the cholesterol side chain (Hamlyn et al., 2003; Murrell et al., 2005; Tripodi et al., 2009).

### 1.2 Endogenous ouabain and hypertension

Dahl et al. (1967) related the elevation of arterial blood pressure to a diet rich in Na^+^, since salt could stimulate a humoral factor with an important role in the pathogenesis of arterial hypertension in rats. In addition to the identification of a Na^+^ transport inhibitor with natriuretic activity in the plasma and urine of humans and dogs (Favre et al., 1975), the evidence of the role played by NCX in smooth vascular muscle and its direct correlation with NKA activity favoured the hypothesis of a ‘natriuretic hormone’ involved in the regulation of blood pressure (Blaustein, 1977). This hypothesis gained strength when Hamlyn et al. (1982) observed that changes in Na^+^ metabolism increased the concentration of an endogenous NKA inhibitor that would be responsible for the increase in peripheral vascular resistance in primary hypertension. This hormone has now been recognized as EO (Hamlyn et al., 1991; Pavlovic, 2020).

Variations in the Na^+^ content of the diet affect hormonal mediators such as Ang II/aldosterone and dopamine and modulate the activity of NKA in the renal tubule. Stimulation of NKA enzymatic activity increases Na^+^ transport through the epithelium of the proximal renal tubule, promoting Na^+^ retention, while inhibition of NKA activity leads to natriuresis. Interestingly, diets rich in Na^+^ also increase the release of endogenous compounds such as EO, which may inhibit NKA activity (especially the plasmerosome‐bearing α2 isoform), increasing intracellular concentrations of Na^+^ ([Na^+^]_i_) and Ca^2+^ ([Ca^2+^]_i_) in vascular smooth muscle cells, and thus the vascular tone, with a corresponding increase in blood pressure (Jaitovich & Bertorello, 2010; Juhaszova & Blaustein, 1997). Another possibility is that ouabain, via the activation of caveolar NKA‐mediated signalling, stimulates the concerted endocytosis of the nephron tubular basolateral NKA α1 and apical Na^+^/H^+^ exchanger‐3 (NHE3), virtually inhibiting the vectorial transport (reabsorption) of Na^+^ (Yan et al., 2016)

The regulatory mechanisms of Na^+^ transport classically considered important for the renal control of Na^+^ homeostasis and blood pressure involve the aldosterone mineralocorticoid receptors (MR), Na^+^‐sensitive epithelial channels (ENaC) and NKA, which are also present in the central nervous system (CNS). A diet rich in Na^+^ promotes an increase in the concentration of Na^+^ in the cerebrospinal fluid (CSF), generating sympathoexcitation and hypertension in salt‐sensitive rats. Evidence indicates that this activates a signalling cascade via a CTS (EO) that is initiated with the binding of aldosterone to MR and upregulates ENaC, increasing the secretion of EO by the hypothalamus, the latter decreasing the electrical potential of the neuronal membrane, leading to greater release of Ang II to act on its receptor (AT1R) (Blaustein et al., 2012; Leenen et al., 2017; Lu et al., 2017). Persistent activation of such a MR–ENaC–EO pathway results in EO‐induced protein expression of angiotensin‐converting enzyme (ACE), AT1R and NADPH oxidase subunits and reduction of neuronal nitric oxide (NO) synthase (Leenen et al., 2020).

Since plasma levels of EO increase in some conditions, such as acute and chronic hypervolaemia, in some animal models of hypertension (Sekihara et al., 1992; Yuan et al., 2000), in congestive heart failure (Blaustein, 2012; Pulgar et al., 2013) and in humans with essential hypertension (Linde et al., 2012), studies have been carried out to understand whether there is a relationship between EO and arterial hypertension using the model of hypertension induced by exogenous ouabain (Briones et al., 2009; Hao et al., 2018; Rossoni et al., 2006).

Although several studies showed that ouabain induces elevated blood pressure, some studies have demonstrated important variations in the pressor response to ouabain in the same experimental group. Moreover, other studies revealed that the administration of ouabain does not promote any alteration of blood pressure compared to control groups. Variables such as species, dose, duration, route of administration and method of measuring blood pressure might influence the response of ouabain, and this review aims to examine the available literature in rats. The tables shown here were adapted and expanded from those originally presented by Ghadhanfar et al. (2014), incorporating recent findings to provide a more comprehensive overview, including additional parameters from subsequent studies.

### 1.3 Ouabain‐induced hypertension

Experimental models allow a better understanding of aetiology, pathophysiology and treatment of the various clinical conditions existing in humans (Choudhary et al., 2018; Leong et al., 2015). However, hypertension is considered multifactorial, so the choice of animal model is essential to obtain reliable results that allow translation for humans. No animal model will fully mimic the mechanisms observed in human hypertension, but depending on the research objective, some species are preferable to others to represent different types of hypertension (Leong et al., 2015; Sarikonda et al., 2009). The administration of exogenous ouabain has been used as a model to study its effect on blood pressure, trying to mimic the possible consequences of endogenous CTS.

Although it is not the best species for studying hypertensive conditions, the rat is one of the most common species used worldwide. Usually, male albino rats of Wistar or Sprague–Dawley strains are predominant, with some reports using Wistar–Kyoto rats, (stroke‐prone) spontaneously hypertensive rats (SHR), or Dahl rats. In general, the studies show that ouabain per se – or its derivatives (Manunta, Hamilton, Hamlyn, 2001) – seems to be mildly hypertensive. The usual elevation of blood pressure in rats is 15–25%, which is not markedly dose‐dependent (Yuan, Manunta, Hamlyn et al., 1993). Data are summarized in Table 1.

**TABLE 1:** Summary of studies demonstrating positive hypertensive effects of exogenous ouabain in rats.

Strain, sex	Age, weight	BP reading method	Dose	Duration	Route of ouabain administration	BP effect	References
SD, male	300–350 g	Arterial catheter	1.5 mg/kg/day	4 weeks	s.c. osmotic pump	↑ 15%	Doursout et al. (1992)
Wistar, male	150–200 g	Tail cuff	17 µg/kg loading dose + 13.9 µg/kg/day + 34 µg/kg loading dose + 27.8 µg/kg/day	4 weeks + 4 weeks	i.p. bolus	↑ 20%	Yuan, Manunta, Chen et al. (1993)†
Wistar, male	350–400 g	Tail cuff Arterial catheter	17 µg/kg loading dose + 13.9 µg/kg/day + 34 µg/kg loading dose + 27.8 µg/kg/day	4 weeks + 4 weeks	i.p. bolus (stepped)	↑ 20%	Yuan, Manunta, Hamlyn et al. (1993)†
			34 µg/kg loading dose + 27.8 µg/kg/day	6 weeks	i.p. bolus (single daily)	↑ 20%	
Wistar, male	300–350 g	Tail cuff	34 µg/kg loading dose + 27.8 µg/kg/day	6 weeks	i.p. bolus	↑ 15%	Pamnani et al. (1994)†
Wistar, male	200–250 g	Arterial catheter	10 µg/day 10 µg/day 25–75 µg/day	2 weeks	i.c.v. infusion i.v. infusion s.c. pellet	↑ 20% ↑ 20% ↑ 30% (HS)	Huang et al. (1994)*
SD, male	180–200 g	Arterial catheter	27.8 µg/kg/day	6 weeks	i.p. bolus	↑ 30%	Kurashina et al. (1996)
SD, male	150–180 g	Tail cuff	50 µg/kg/day	10 weeks	s.c. osmotic pump	↑ 15%	Quadri et al. (1997)†
SD, male	7–11 weeks old, 200–250 g	Tail cuff Arterial catheter	23.75 µg/kg/day	6 weeks	i.p. bolus	↑ 15–20%	Wang et al. (1997)‡
Wistar, male	7‐8 weeks old, 200–250 g	Arterial catheter	75 µg/day	12 days	s.c. pellet	↑ 20% (+HS)	Huang et al. (1999)*
Wistar, male	150–200 g	Arterial catheter	50 µg/day	2 weeks	s.c. osmotic pump	↑ 20%	Huang and Leenen (1999)*
Wistar, male	150–200 g	Arterial catheter	50 µg/day	3 weeks	s.c. pellet	↑ 20%	Veerasingham and Leenen (1999)*
Wistar, male	150–200 g	Arterial catheter	50 µg/day	2 weeks	s.c. osmotic pump	↑ 25%	Veerasingham et al. (2000)*
SD, male	7–11 weeks old, 200–250 g	Tail cuff	20 µg/kg/day	5 weeks	i.p. bolus	↑ 15%	Wang et al. (2000)‡
SD, male	3 weeks old	Tail cuff Arterial catheter	10 µg/mL (758 ± 61 µg/kg/day)	12 weeks	Oral (drinking water)	↑ 10–15%	Tamura et al. (2000)
SD, male	200–250 g	Tail cuff Arterial catheter	15 µg/kg/day	5 weeks	s.c. osmotic pump	↑ 20%	Kimura et al. (2000)†
SD, male	7–8 weeks old	Arterial catheter	30 µg/kg/day	5 weeks	s.c. osmotic pump	↑ 30%	Manunta et al. (2000)†
		Tail cuff	15 µg/kg/day	6 weeks	s.c. osmotic pump	↑ 15%	
SD, male	7–8 weeks old	Tail cuff Arterial catheter	25 µg/kg/day	5 weeks	s.c. osmotic pump	↑ 35%	Manunta, Hamilton, Hamlyn (2001)†
SD, male	7‐11 weeks old, 200–250 g	Tail cuff	20 µg/kg/day	6 weeks	i.p. bolus	↑ 15%	Wang et al. (2001)§
Wistar, male	200–250 g	Arterial catheter	50 µg/day	2 weeks	s.c. osmotic pump	↑ 25%	Zhang and Leenen (2001)*
Wistar, male	6 weeks old	Tail cuff	25 µg/day	5 weeks	s.c. pellet	↑ 25%	Rossoni, Salaices, Marín et al. (2002); Rossoni, Salaices, Miguel et al. (2002)¶
SD, male	250–300 g	Arterial catheter	7, 14 or 28 µg/kg/day	4 weeks	s.c. osmotic pump	Up to ↑ 60% No change (7)	Di Filippo et al. (2003)
SD, male	7–8 weeks old	Tail cuff	30 µg/kg/day	5 weeks	s.c. osmotic pump	↑ 30%	Iwamoto et al. (2004)
Wistar, male	6 weeks old	Tail cuff	8 µg/day	5 weeks	s.c. pellet	↑ 25%	Xavier, Rossoni, et al. (2004)¶
Wistar, male	6 weeks old	Arterial catheter	8 µg/day	5 weeks	s.c. pellet	↑ 25%	Xavier, Salaices et al. (2004)¶
Wistar, male	6 weeks old	Tail cuff	8 µg/day	5 weeks	s.c. pellet	↑ 30%	Xavier, Yogi et al. (2004)¶
Wistar, male	150–200 g	Arterial catheter	50 µg/day	2 weeks	s.c. osmotic pump	↑ 20%	Kent et al. (2004)*
		Arterial catheter	10 µg/day	2 weeks	i.c.v. infusion	↑ 20%	
SD, male	3 weeks old, 100–110 g	Tail cuff	15 µg/kg/day	18 weeks	s.c. osmotic pump	↑ 15%	Ferrandi et al. (2004)†
SD, male	180–220 g	Tail cuff	27.8 µg/kg/day	6 weeks	i.p. bolus	↑ 45%	Ge et al. (2005)§
Wistar, male	150–200 g	Arterial catheter	50 µg/day	2 weeks	s.c. osmotic pump	↑ 15%	Cheung et al. (2006)*
SD, male	80–100 g	Tail cuff	27.8 µg/kg/day	8 weeks	i.p. bolus	↑ 45%	Ge & Lü (2006)§
SD, male	80–100 g	Tail cuff	34 µg/kg loading dose + 27.8 µg/kg/day	6 weeks	i.p. bolus	↑ 20%	Jiang Guo, Lü (2006)§
Wistar, male	6 weeks old	Arterial catheter	8 µg/day	5 weeks	s.c. pellet	↑ 15%	Rossoni et al. (2006)¶
Wistar, male	6 weeks old	Tail cuff	8 µg/day	5 weeks	s.c. pellet	↑ 25%	Briones et al. (2006)¶
Wistar, male	6 weeks old	Tail cuff	8 µg/day	5 weeks	s.c. pellet	↑ 15%	Hernanz et al. (2008)¶
Wistar, male	6 weeks old	Tail cuff	8 µg/day	5 weeks	s.c. pellet	↑ 25%	Aras‐Lópes et al. (2008)¶
Wistar, male	8–12 weeks old	Arterial catheter	25 µg/kg/day	3, 7, 15 or 30 days	s.c. injection in soy oil	↑ 25% (after 15 days)	Padilha et al. (2008a)¶
Wistar, male	10 weeks old	Arterial catheter	25 µg/kg/day	2 weeks	s.c. injection in soy oil	↑ 15%	Padilha et al. (2008b)¶
Wistar, male	6 weeks old	Tail cuff	8 µg/day	5 weeks	s.c. pellet	↑ 15%	Briones et al. (2009)¶
SHR, male	6 weeks old	Arterial catheter	8 µg/day	5 weeks	s.c. pellet	↑ 20%	Xavier et al. (2009)¶
SD, male	500–600 g	Tail cuff	25‐30 µg/kg/day	5 weeks	s.c. pellet	↑ 10%	Zhang et al. (2009)†
SD, male	250 g	Tail cuff	30 µg/kg/day	5 weeks	s.c. pellet	↑ 30%	Cao et al. (2009)†
SD, male	ND	Tail cuff	25 µg/day? (25 µg/kg/day in the abstract)	5 weeks	s.c. pellet	↑ 25%	Pulina et al. (2010)†
SD, male	180–200 g	Tail cuff	27.8 µg/kg/day	4 weeks	i.p. bolus	↑ 30%	Ge et al. (2010)§
SD, male	7‐9 weeks old, 160–200 g	Tail cuff	34 µg/kg loading dose + 27.8 µg/kg/day	5 weeks	i.p. bolus	↑ 25%	Zhang et al. (2010)§
SD, male	200‐250 g	Arterial catheter	1 µg/kg/day	8 days	i.p. bolus	↑ 25%	Holthouser et al. (2010)
Wistar, male	6 weeks old	Tail cuff	8 µg/day	5, 10 or 20 weeks	s.c. pellet	↑ 25%	Wenceslau et al. (2011)¶
WKY, male	5 or 45 weeks old	Tail cuff	8.3 µg/day	7 weeks	s.c. pellet	↑ 20%	Silva et al. (2011)
SD, male	6‐7 weeks old, 180–220 g	Tail cuff	27.8 µg/kg/day	5 weeks	i.p. bolus	↑ 20%	Liu et al. (2012)
SD, male	6‐7 weeks old, 180–220 g	Tail cuff	27.8 µg/kg/day	5 weeks	i.p. bolus	↑ 15%	Cui et al. (2012)
SD, male	6‐8 weeks old, 160–200 g	Tail cuff	34 µg/kg loading dose + 27.8 µg/kg/day	6 weeks	i.p. bolus	↑ 15%	Zhao et al. (2013)
SD, male	12 weeks old	Tail cuff	25 µg/day	8 weeks	s.c. pellet	↑ 30%	Pulgar et al. (2013)
SD, male	ND	ND	30 µg/kg/day	7 weeks	ND	↑ 30%	Liu et al. (2014)
SD, male	200–250 g	Arterial catheter	1 µg/kg/day	9 days	i.p. bolus	↑ 20%	Khundmiri et al. (2014)
SD, male	5 weeks old, 110–120 g	Tail cuff	15 µg/kg/day	12 weeks	s.c. osmotic pump	↑ 15%	Ferrandi et al. (2014)†
Wistar, male	6 weeks old	Arterial catheter	8 µg/day	5 or 20 weeks	s.c. pellet	ND	Davel et al. (2014)¶
Wistar, male	12 weeks old	Arterial catheter	25 µg/kg/day	2 weeks	s.c. injection in soy oil	↑ 15%	Meira et al. (2015)¶
SD, male	120–150 g	Tail cuff	30 µg/kg/day	8 weeks	s.c. osmotic pump	↑ 35%	Villa et al. (2016)†
SHR, male	45 days old	Tail cuff	30 µg/kg/day	5 weeks	s.c. injection in soy oil	↑ 8%	Oliveira et al. (2021)¶
Wistar, male	45 days old	Tail cuff	30 µg/kg/day	5 weeks	s.c. injection in soy oil	↑ 15%	França‐Neto et al. (2022)¶
SD, male	160–190 g	Tail cuff	27.8 µg/kg/day	8 weeks	i.p. bolus	↑ 25%	Tang et al. (2024)

*n* ranged from 4 to 35 (controls and ouabain‐treated rats; in some works it was not described). The symbols in the references represent publications from the same or affiliated research groups. BP, blood pressure; Dahl R, salt‐resistant rats; Dahl S, salt‐sensitive rats; DBP, diastolic BP; HS, high salt diet; i.c.v., intracerebroventricular; i.v., intravenous; ND, not described; s.c., subcutaneous; SD, Sprague–Dawley; SHR, spontaneously hypertensive rats; WKY, Wistar Kyoto.

To achieve steady plasma concentration, subcutaneous (s.c.) administration (by osmotic minipumps, controlled time‐release pellets, or daily injections) has been the preferred route for a slow, stable delivery of ouabain, as well as daily intraperitoneal (i.p.) bolus injections and intravenous (i.v.) infusion. The drinking water was used as an oral route in one study. Tamura et al. (2000) measured the blood pressure (tail cuff and arterial catheter) of male Sprague–Dawley rats raised for 12 weeks drinking water with 10 mg/mL ouabain (around 750 µg/kg/day), and detected a significant elevation compared to controls (10–15%) as well as an elevation in plasma aldosterone and K^+^. Furthermore, an i.v. bolus was employed for an immediate, short‐term cardiovascular effect, and intracerebroventricular (i.c.v.) acute administration was used to unveil the CNS role of ouabain in cardiovascular function.

In these studies, the dose for s.c. administration ranged from 10 to ∼200 µg/kg/day, and at least 2 weeks was necessary to achieve a significant increase of the systolic blood pressure (or mean blood pressure) independent of the dose, though sometimes much more time was needed (slow‐release s.c. pellets delivering 25 µg/day significantly raised systolic blood pressure after the seventh week – Pulgar et al., 2013). Lower doses gave variable results. Di Filippo et al. (2003) showed that 7 µg/kg/day (4 weeks) was not enough to produce the elevation of mean blood pressure. However, Manunta et al. (1994), using 3 µg/kg/day (5 weeks), exhibited a significant increase of mean blood pressure (but not systolic blood pressure). Apparently, there is no drug tolerance since the hypertensive effect lasts up to 20 weeks of constant administration (Wenceslau et al., 2011). On the other hand, the effect subsides after the administration stops (Manunta et al., 1994).

For the i.p. administration, the studies used a shorter range (15–30 µg/kg/day), and, in contrast to the s.c. administration, two works from the same group used a very low dose (1 µg/kg/day) for 8–9 days (Holthouser et al., 2010; Khundmiri et al., 2014). One work using i.v. infusion administered around 40 µg/kg/day for 2 weeks (Huang et al., 1994). In the case of the i.c.v. administration, bolus injections ranged from very low doses (0.00006 µg, Fedorova et al., 2007; 0.001 µg, Teruya et al., 1997) up to 1 µg, a dose reported to affect rat behaviour, evoking back‐and‐forth movements, and some variations in blood pressure. This was more intense with 3 µg (Huang & Leenen, 1992). Huang et al. (1994) and Kent et al. (2004) conducted an i.c.v. infusion of 10 µg/day for 2 weeks.

Comparing different routes of administration, Huang et al. (1994) administered ouabain chronically through an i.c.v., i.v. (both delivering 10 µg/day by minipumps) or s.c. route (25 µg/day using pellets) in male Wistar rats, fed with regular or high salt diet for up to 2 weeks, and observed an increase of 20–30 mmHg in all groups. This seemed to be dose‐dependent, as 75 µg/day (three s.c. pellets) resulted in a larger blood pressure elevation compared to one pellet. The hypertensive effect was antagonized by the ganglionic blocker hexamethonium or a vasopressin antagonist.

#### 1.3.1 Mechanisms of ouabain hypertensinogenic effect

**FIGURE 2:**
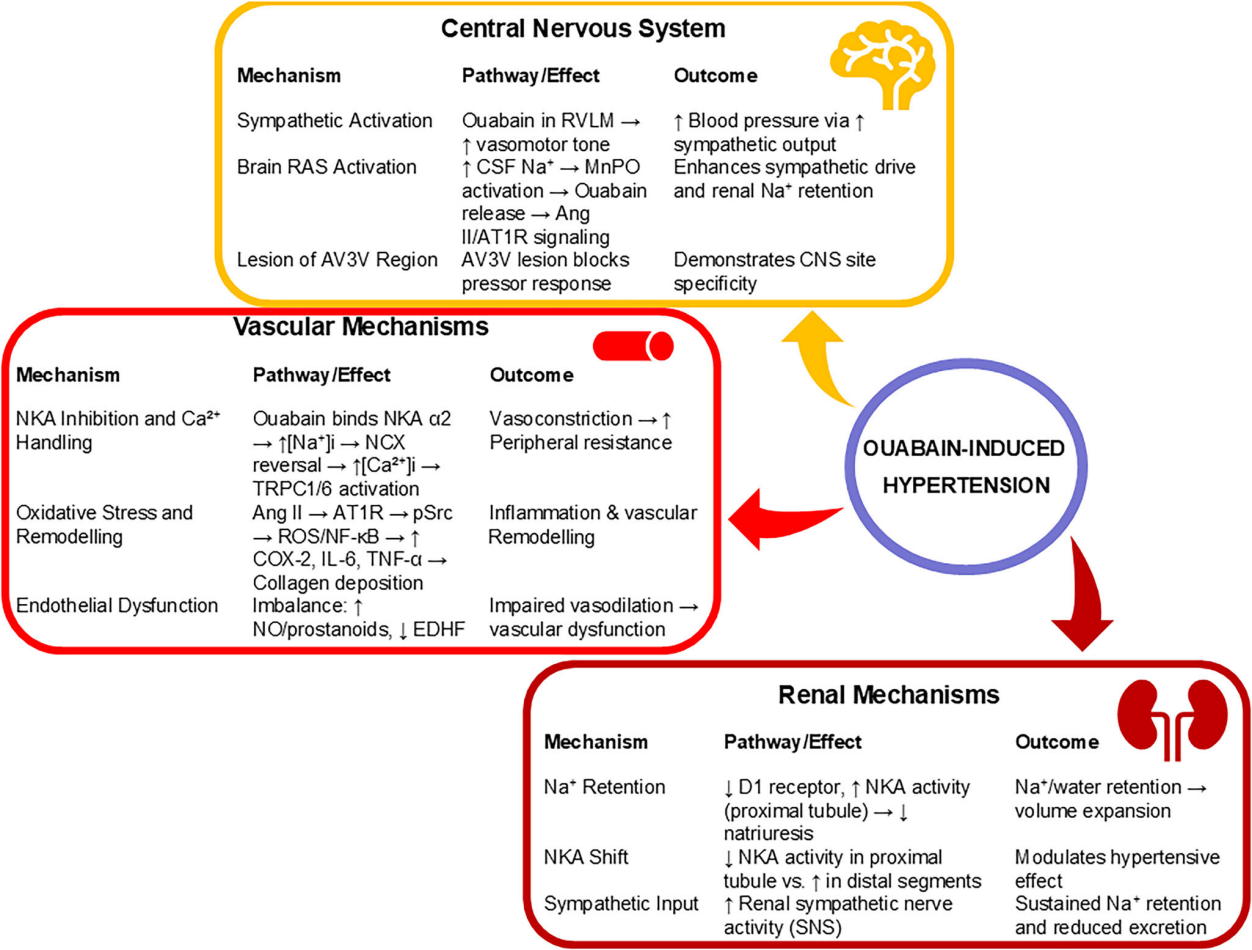
Mechanisms, pathways and outcomes associated to ouabain‐induced hypertension. Peripheral and central effects of ouabain are responsible for blood pressure modulation and may contribute to the hypertensive state in the long‐term. Ang II, angiotensin II; AT1R, angiotensin II type 1 receptor; AV3V, anteroventral third ventricle; CNS, central nervous system; COX, cyclooxygenase; CSF, cerebrospinal fluid; D1, dopamine D1; EDHF, endothelial‐derived hyperpolarizing factor; IL‐6, interleukin 6; MnPO, median preoptic nucleus; NCX, Na^+^/Ca^2+^‐exchanger; NF‐κB, nuclear factor κB; NKA, Na^+^/K^+^‐ATPase; NO, nitric oxide; PVN, paraventricular nucleus; RAS, renin–angiotensin system; RVLM, rostral ventrolateral medulla; SNS, sympathetic nervous system; TNF, tumour necrosis factor α; TRPC, transient receptor potential canonical channels.

Ouabain‐induced hypertension is a complex phenomenon involving multiple mechanisms at both cellular and systemic levels (illustrated in Figure 2). Some of these mechanisms include inhibition of NKA and Na^+^ handling – by such a classically acknowledged molecular mechanism, the accumulation of [Ca^2^⁺]_i_ enhances vascular smooth muscle contraction and increases peripheral resistance (Yuan, Manunta, Hamlyn et al., 1993), a key contributor to elevated blood pressure. Iwamoto et al. (2004) showed that ouabain‐induced hypertension in SD rats (30 µg/kg/day for 5 weeks) was reduced by a selective NCX inhibitor that preferentially blocks the Ca^2+^ entry mode (SEA0400) as well as the vasoconstriction and rise of [Ca^2+^]_i_ by nanomolar ouabain. Higher resting [Ca^2+^]_i_ and phenylephrine‐induced Ca^2+^ transients are accompanied by increased expression of the ouabain‐sensitive NKA α2‐subunit, NCX1 and components of Ca^2+^ entry pathways, such as the transient receptor potential channels (TRPC1 and TRPC6), leading to augmented store‐operated and receptor‐operated Ca^2+^ entry in mesenteric arteries, which may underlie the heightened vasoconstrictive responses (Pulina et al., 2010).

Ouabain‐induced hypertension is associated with structural and functional vascular changes. These include increased collagen deposition and inward hypotrophic remodelling in third‐order resistance arteries, contributing to increased vascular stiffness (Briones et al., 2006; Zhang et al., 2009). Although normal responses to noradrenaline and acetylcholine are usually observed (Xavier, Rossoni et al., 2004; Xavier, Salaices et al., 2004; Xavier, Yogi et al., 2004), quite long ouabain treatment (20 weeks) provokes enhanced noradrenaline‐induced contractions (Wenceslau et al., 2011).

Oxidative stress and inflammation play key roles, as evidenced by increased reactive oxygen species (ROS) production, cyclooxygenase (COX)‐2 expression, and elevated proinflammatory cytokines such as interleukin (IL)‐6 and tumour necrosis factor (TNF)‐α in mesenteric resistance arteries (de Oliveira et al., 2021; França‐Neto et al., 2022; Wenceslau et al., 2011), but not in all vascular beds (Briones et al., 2009; Hernanz et al., 2008). Endothelial modulation is altered. In the thoracic aorta, increased endothelial and neuronal NO synthase activity, along with the release of hyperpolarizing factors, may act as counter‐regulatory mechanisms to mitigate the hypertensive effects of ouabain (Rossoni, Salaices, Marín et al., 2002; Rossoni, Salaices, Miguelet al., 2002). However, in mesenteric resistance arteries, ouabain increases the release of NO and prostanoids but impairs endothelium‐derived hyperpolarizing factor (EDHF), alongside increased COX‐2 expression, contributing to sustained hypertension (Aras‐López et al., 2008). In contrast, Cui et al. (2012) showed a decrease in aortic NO production, and Liu et al. (2012) in the thoracic aorta. Furthermore, even before a significant increase of blood pressure, the same group showed that the sensitivity to endothelin‐1 (ET‐1) and its expression as well as of its receptors was higher in ouabain‐treated arteries, and also left ventricular enlargement, cardiac wall thickening, and myocardial ultrastructural alterations (Jiang, Guo, Lü, 2006; Jiang, Guo, Lü, Ren, 2006; Liu et al., 2012).

Additionally, ouabain hypertension is linked to changes in NKA activity and α‐isoform protein expression, with regional variations in vasopressor responses and enzymatic regulation (Rossoni, Salaices, Marín et al., 2002; Rossoni, Salaices, Miguelet al., 2002). Pulgar et al. (2013) showed that NKA activity was decreased in mesenteric resistance arteries, whereas NKA α2 isoform protein expression was unchanged. The Ang II–AT1R–pSrc–ROS–nuclear factor κB (NF‐kB)–COX‐2 signalling pathway drives small‐artery remodelling via extracellular matrix changes and apoptosis, independent of haemodynamic factors (França‐Neto et al., 2022).

Some studies have highlighted the critical role of renal mechanisms in the development of ouabain‐induced hypertension in normotensive rat models. Kurashina et al. (1996) suggested that impairment of renal pressure‐natriuresis was a primary mechanism contributing to ouabain's hypertensive effect. This renal‐centric view was reinforced by Zhang et al. (2010), who demonstrated that 5 weeks of ouabain infusion resulted in a significant increase in systolic blood pressure from the fourth week onward, associated with nearly fourfold elevated renal ouabain concentrations. This was accompanied by decreased fractional Na^+^ excretion, downregulation of the D1 dopamine receptor and increased renal NKA activity (and also Holthouser et al., 2010), all indicative of altered tubular Na^+^ handling as a hypertensive mechanism. Similarly, Ge et al. (2005) and Ge and Lü (2006) observed that although systolic blood pressure remained unchanged during the first 2 weeks of ouabain administration, it rose significantly after week 4. Their data showed a shift in Na^+^ reabsorption from post‐proximal to proximal nephron segments, suggesting proximal tubular Na^+^ retention as a key contributor to hypertension. These changes occurred independently of sympathetic activity, as confirmed in later work (Ge et al., 2010). On the other hand, Silva et al. (2011) reported unchanged Na^+^ and K^+^ urinary excretion, and thus the hypertensive effect of ouabain was independent of its effects on renal NKA, since lower activity in the proximal tubules (in young but not in aged rats) was possibly balanced by distal segments. Tang et al. (2024) showed that renal sympathetic denervation impaired the development of ouabain‐induced hypertension. Thus, these findings suggest that hypertension involves altered renal Na^+^ handling, particularly in proximal tubules, but may also depend on distal compensation and sympathetic activity.

#### 1.3.2 Central nervous system mechanisms

Ouabain‐induced hypertension involves CNS processes, particularly through its effects on sympathetic activation and modulation of the brain renin–angiotensin system (RAS). Research has shown that i.c.v. administration of ouabain raises blood pressure by increasing sympathetic outflow, impairing baroreflex control, and interacting with brain Na^+^ levels (Table 2).

**TABLE 2:** Summary of studies demonstrating positive hypertensive effects of exogenous ouabain administered in the central nervous system of rats.

Strain, sex	Age, weight	BP reading method	Dose	Route of ouabain administration	BP effect	References
Wistar/WKY/SHR, male	16 weeks old, 250–300 g	Arterial catheter	1, 10 or 100 µg	i.c.v. bolus	Up to ↑ 30%	Takahashi, Iyoda, Takeda, Okajima et al. (1984)‡
Wistar, male	12 weeks old, 265 ± 5 g	Arterial catheter	0.01–10 µg	i.c.v. bolus	Up to ↑ 30%	Takahashi, Iyoda, Takeda, Sasaki et al. (1984)‡
SD, male	200–250 g	Arterial catheter	80 µg/kg	i.c.v. bolus	↑ 50% (DBP)	Caldwell et al. (1985)
Wistar, male	8 weeks old, 218 ± 5 g	Arterial catheter	0.01, 0.1 or 1 µg	i.c.v. bolus	Up to ↑ 20%	Iyoda et al. (1986)‡
Wistar, male	9 weeks old	Arterial catheter	0.01, 0.1, 1 or 10 µg	i.c.v. bolus	Up to ↑ 30%	Takahashi et al. (1987)‡
Wistar, male	285–440 g	Arterial catheter	20 ng	i.c.v. bolus	Up to ↑ 30%	Jones and Lo (1990)
SD, male	250–300 g	Arterial catheter	0.3 µg	i.c.v. bolus	↑ 10%	Shah and Jandhyala (1991)
WKY, male	3.5 weeks old	Arterial catheter	0.1, 0.3 or 1 µg	i.c.v. bolus	Up to ↑ 15%	Huang and Leenen (1992)*
Wistar, male	180–200 g	Arterial catheter	0.3 or 1 µg	i.c.v. bolus (after AVP antagonist i.v.)	Up to ↑ 20%	Huang et al. (1992)*
WKY, male	8 weeks old	Arterial catheter	0.1, 0.3 or 1 µg	i.c.v. bolus	ND	Leenen et al. (1993)*
Dahl S/Dahl R, male	7 weeks old	Arterial catheter	0.3 or 1 µg	i.c.v. bolus	Up to ↑ 30%	Huang et al. (1994)*
WKY, male	5–9 weeks old	Arterial catheter	0.1, 0.3 or 1 µg	i.c.v. bolus	Up to ↑ 15%	Huang and Leenen (1995)*
SHR, male	9 weeks old	Arterial catheter	0.3 or 0.6 µg	i.c.v. bolus	Up to ↑ 15%	Huang and Leenen (1996a)*
Wistar, male	200–220 g	Arterial catheter	0.3 or 0.6 µg	i.c.v. bolus	Up to ↑ 20%	Huang and Leenen (1996b)*
Wistar, male	175–200 g	Arterial catheter	0.3 or 0.6 µg	i.c.v. bolus	Up to ↑ 20%	Huang et al. (1997)*
Wistar, male	288 ± 3 g	Arterial catheter	0.1 µg	i.c.v. bolus	↑ 10%	Budzikowski and Leenen (1997)*
Wistar, male	150‐200 g	Arterial catheter	0.3 or 0.6 µg	i.c.v. bolus	Up to ↑ 15%	Veerasingham and Leenen (1997)*
SD, male	7–8 weeks old, 240–340 g	Arterial catheter	1, 10 or 100 ng	i.c.v. bolus	Up to ↑ 25%	Teruya et al. (1997)
Dahl S/Dahl R, male	9 weeks old	Arterial catheter	0.5 µg	i.c.v. bolus	↑ 20–25%	Huang and Leenen (1998)*
Wistar, male	4.5 weeks old	Arterial catheter	0.05, 0.1, 0.2 or 0.4 µg	i.c.v. bolus	↑ 15%	Budzikowski and Leenen (2001)*
SD, male	300–350 g	Arterial catheter	0.3 or 0.6 µg	i.c.v. bolus	Up to ↑ 25%	Huang, Ganten et al. (2001)*
Dahl S/Dahl R, male	7–8 weeks old	Arterial catheter	0.5 µg	i.c.v. bolus	↑ 20% (+HS)	Huang, Wang et al. (2001)*
SD, male	250–300 g	Arterial catheter	3 or 6 µg	i.c.v. bolus (PAG)	Up to ↑20%	D'Amico et al. (2003)
Dahl S, male	10 weeks old	Arterial catheter	60 pg	i.c.v. bolus	↑ 35%	Fedorova et al. (2007)

*n* ranged from 5 to 65 (controls and ouabain‐treated rats; in some works, it was not described). The symbols in the references represent publications from the same or affiliated research groups. AVP, atrial natriuretic peptide/vasopressin; BP, blood pressure; Dahl R, salt‐resistant rats; Dahl S, salt‐sensitive rats; DBP, diastolic BP; HS, high salt diet; i.c.v., intracerebroventricular; i.v., intravenous; PAG, periaqueductal grey area; SHR, spontaneously hypertensive rats; SD, Sprague–Dawley; WKY, Wistar Kyoto.

Blood‐borne aldosterone, ouabain and Ang II primarily exert their central effects in regions adjacent to the circumventricular organs (CVOs) of the third and fourth ventricles, where the blood–brain barrier is weak or absent. These substances influence key hypothalamic and brainstem nuclei involved in long‐term blood pressure regulation, including the subfornical organ (SFO), paraventricular nucleus (PVN) and supraoptic nucleus (SON). Both circulating and locally synthesized EO have been shown to act in these areas (Leenen et al., 2020). Additionally, posterior hypothalamus ouabain induced blood pressure elevation (Iyoda et al., 1986). Other regions implicated in the central control of blood pressure include the periaqueductal grey area (D'Amico et al., 2003), the anteroventral third ventricle (AV3V) area (Takahashi, Iyoda, Takeda, Okajima et al., 1984; Takahashi, Iyoda, Takeda, Sasaki et al., 1984; Veerasingham & Leenen, 1997) and the organum vasculosum of the lamina terminalis (OVLT), which extends into the ventral median preoptic nucleus (MnPO) – both critical sites for integrating neurohumoral signals that influence sympathetic outflow and fluid balance (Budzikowski & Leenen, 1997, 2001; Veerasingham & Leenen, 1997, 1999).

One of the key mechanisms involves sympathetic activation (Yuan, Manunta, Hamlyn et al., 1993). Studies have demonstrated that ouabain infusion into the third ventricle induces seizures in conscious rats but leads to hypertension in anaesthetized ones (Jacomini et al., 1984). Additionally, i.c.v. ouabain has been found to blunt the hypotensive effect of i.c.v. K^+^, suggesting an increase in central sympathetic drive (Shah & Jandhyala, 1995). Microinjection of ouabain into the rostral ventrolateral medulla (RVLM) of anesthetized normotensive rats evokes tonic activity of vasomotor neurons and also induces hypertension at least partially through M2 muscarinic receptors, an effect that can be counteracted by digoxin‐specific antibodies (Teruya et al., 1997). Veerasingham and Leenen (1997) demonstrated that in rats with systemic arginine vasopressin blockade, a discrete area of the vAV3V region is involved in mediating part of the pressor responses to i.c.v. Na^+^ and ouabain but not to Ang II. Furthermore, excitotoxic lesions in the AV3V prevented ouabain‐induced hypertension, emphasizing the critical role of this region in blood pressure regulation (Veerasingham & Leenen, 1999). In the peripheral ganglia, ouabain enhances long‐term potentiation, increasing preganglionic sympathetic nerve activity (Aileru et al., 2001). This amplification of sympathetic output is particularly evident in the PVN, where brain‐specific immunoneutralization of ouabain prevents hypertension and sympathetic hyper‐reactivity in rats chronically infused with peripheral ouabain (Huang et al., 1994).

Another important factor is ouabain's interaction with brain Na^+^ and Ang II. Acute i.c.v. administration of 0.1, 0.3 or 1 µg ouabain increases blood pressure in Wistar Kyoto (WKY) rats and SHRs, but this response is attenuated by chronic high Na^+^ intake, suggesting a complex relationship between Na^+^ and EO (Leenen et al., 1993). In normotensive rats, elevated CSF Na^+^ has been shown to raise blood pressure by triggering brain ouabain release in the MnPO, linking Na^+^ sensitivity to hypertension through Ang II/ATR1 (Budzikowski & Leenen, 1997, 2001). Moreover, ouabain‐induced sympathetic hyperactivity in Dahl salt‐sensitive rats is prevented by central Ang II receptor blockade, indicating that Ang II signalling in the brain occurs downstream of ouabain (Huang & Leenen, 1998). Using transgenic rats deficient in brain angiotensinogen, Huang, Ganten et al. (2001) and Huang, Wang et al. (2001) showed a clear decrease in sympathoexcitatory and pressor responses to CSF Na^+^ and i.c.v. ouabain.

Chronic exposure to ouabain further supports its central role in hypertension. Continuous s.c. infusion of ouabain at 50 µg/day leads to an increase in blood pressure, coinciding with elevated hypothalamic Ang II levels and suppression of the circulatory and renal RAS (Cheung et al., 2006). Importantly, this ouabain‐induced hypertension is prevented by peripheral administration of ATR1 antagonists, possibly by central blockade, and impairment of central baroreceptor reflex was also suggested (Huang & Leenen, 1999; Zhang & Leenen, 2001). Moreover, intrahippocampal ouabain mimics the effects of NaCl loading, increasing blood pressure and natriuresis through marinobufagenin, a bufadienolide NKA inhibitor (Fedorova et al., 2007).

### 1.4 Resistance to ouabain‐induced hypertension

Although the previously mentioned studies have observed elevation of blood pressure through daily administrations of ouabain, others did not observe this phenomenon, highlighting the complexity and variability of this pharmacological model. The absolute number of reports is much less when compared to the ouabain‐induced hypertension ones (Table 3). Still, it can be explained by the fact that the research groups that were successful in achieving ouabain‐induced hypertension persevered in producing a series of studies on this model.

**TABLE 3:** Summary of studies demonstrating no hypertensive effects of exogenous ouabain in rats.

Strain, sex	Age, weight	BP reading method	Dose	Duration	Route of ouabain administration	Observations	References
SD, male	250–300 g	Tail cuff	1, 5 or 10 mg/kg/day	26 days	i.p. injections	Also for 1K‐salt loaded rats	Nirasawa et al. (1985)
SD, male	130–150 g	Tail cuff	1 or 2 mg/week	6 weeks	s.c. injection in sesame oil	Also for 1K rats	Sekihara et al. (1992)
WKY, male	280–290 g, 12–14 weeks old	Arterial catheter	0.001, 0.01 or 0.1 µg/kg/h	9 days (3 days/dose)	i.v. or i.c.v. infusion		Sato and Seto (1993)
SD, male	213–273 g	Tail cuff Arterial catheter	10 or 100 µg/kg/day	4 weeks	i.p. osmotic pump		Li et al. (1995)
Long–Evans, male	350–450 g, 12–24 weeks old	Arterial catheter	30 or 150 µg/kg/day	4 weeks	i.v. infusion	Also for 1K rats	Wang et al. (1999)
SD, male	350–400 g	Tail cuff	50 µg/kg/day	4 weeks	s.c. osmotic pump	Odd vascular reactivity	Cargnelli et al. (2000)
WKY, male	226 ± 10 g	Tail cuff	100 µg/kg/day	16 weeks	s.c. osmotic pump	Adrenal ZG growth	Neri et al. (2006)
SD, male	6 weeks old	Tail cuff	90 µg/kg/day	14 weeks	s.c. osmotic pump	Development of cardiac fibrosis	Kamimura et al. (2012)
Wistar, male	391 ± 21 g, 21–23 weeks old	Telemetric transmission	60 or 320 µg/kg/day	11 weeks	s.c. pellet	Vagal stimulation at inactive period	Ghadhanfar et al. (2014)
SD, male	180–200 g	Tail cuff	27.8 mg/kg/day	4 weeks	i.p. injections		You et al. (2014)

*n* = 3–46 (controls and ouabain‐treated rats). 1K, rats with one kidney; BP, blood pressure; SD, Sprague–Dawley; WKY, Wistar Kyoto; ZG, zona glomerulosa.

Indeed, while several research groups have consistently observed ouabain‐induced hypertension, other studies report neutral responses across a range of rat strains, administration routes, doses and even the presence of digoxin‐like cardiotonic steroids of plant origin in standard rodent chow (Blaustein & Hamlyn, 2024; Ghadhanfar et al., 2014). For example, chronic ouabain infusion in SHRs consistently increases systolic blood pressure (de Oliveira et al., 2021; Xavier et al., 2009), supporting the role of genetic predisposition and heightened sympathetic tone in the pressor response. Conversely, Sprague–Dawley, Wistar, Long‐Evans, and WKY rats may exhibit little to no change in blood pressure, as shown in Table 3, even when protocols and doses are like those used in positive studies.

Several factors were proposed to contribute to this discrepancy. First, genetic strain differences appear to be a determinant. For example, Picotti et al. (1982) and Huang et al. (1994) noted that Wistar rats possess a higher sympathetic tone than Sprague–Dawley rats, which may partly explain their heightened sensitivity to ouabain. Similarly, SHRs respond to ouabain with exaggerated increases in blood pressure and sympathetic activity, whereas normotensive WKY rats do not, suggesting a genetic or neurohumoral component (Aileru et al., 2001; Xavier et al., 2009). The relationship between ouabain and hypertension has been questioned by some investigators due to inconsistent blood pressure responses in outbred rats; however, this variability is now well understood to be largely genetically determined. Studies by Hamlyn and colleagues demonstrated that by selective inbreeding of rats based on their blood pressure response to ouabain, it is possible to generate highly ouabain‐sensitive and ouabain‐resistant strains within just three generations (Aileru et al., 2001; Blaustein, 2018). The sensitive strain showed altered ganglionic synaptic plasticity reversible by in vivo captopril, while resistance was associated with elevated vagal tone and increased levels of calcitonin gene‐related peptide (CGRP) (Aileru et al., 2001; Ghadhanfar et al., 2014; Hamlyn & Blaustein, 2016). Even within the same strain, variability in response is not unusual and parallels findings with other hypertensinogenic agents such as high salt or mineralocorticoids (Table 4). These findings strongly support the conclusion that genetic background and physiology are critical determinants of the pressor effects of ouabain and underscore the importance of considering strain differences in hypertension research.

**TABLE 4:** Summary of studies demonstrating differing levels of sensitivity to the hypertensive effects of ouabain within the same experimental context.

Strain, sex	Age, weight	BP reading method	Dose	Duration	Route of ouabain administration	BP effect	References
SD, male	7–8 weeks old, >230 g	Tail cuff	10, 15, or 30 µg/kg/day 30 µg/kg/day 3 µg/kg/day	5 weeks	s.c. osmotic pump	Up to ↑ 20% *1 out of 8 (no effect)* No effect (SBP)	Manunta et al. (1994)†
SD, male	6‐7 weeks old, 150–180 g	Tail cuff	50 µg/kg/day	10 weeks	s.c. osmotic pump	↑ 15% *20–30% (no effect)*	Ferrari et al. (1998)†
SD, both	200–250 g	Tail cuff	30 µg/kg/day	5 weeks	s.c. osmotic pump	↑ 30% (BOS) *8% (no effect, BOR)*	Aileru et al. (2001)†
SD, male	6‐10 weeks old, 150–200 g	Tail cuff	34 µg/kg loading dose + 27.8 µg/kg/day	6 weeks	i.p. bolus	13 out of 20 ‐ ↑45% *7 out of 20 (no effect)*	Tian et al. (2001)§
SD, male	6‐10 weeks old, 150–200 g	Tail cuff	34 µg/kg loading dose + 27.8 µg/kg/day	8 weeks	i.p. bolus	45 out of 52 ‐ ↑ 30% *7 out of 52 (no effect)*	Ren et al. (2006)§
SD, male	6‐10 weeks old, 150–200 g	Tail cuff	34 µg/kg loading dose + 27.8 µg/kg/day	6 weeks	i.p. bolus	40 out of 48 ‐ ↑20% *8 out of 48 (no effect)*	Jiang et al. (2007)§
SD, male	180–250 g	Tail cuff	20 µg/kg/day	8 weeks	i.p. bolus	10 out of 12 ‐ ↑25% *2 out of 12 (no effect)*	Ren et al. (2013)§

*n* ranged from 6 to 80 (controls and ouabain‐treated rats). The symbols in the references represent publications from the same or affiliated research groups. BOR, Baltimore ouabain‐resistant rats; BOS, Baltimore ouabain‐sensitive rats; BP, blood pressure; i.p., intraperitoneal; SBP, systolic BP; s.c., subcutaneous; SD, Sprague–Dawley; SHR, spontaneously hypertensive rats; WKY, Wistar Kyoto.

Strain‐ and model‐specific hormonal responses, such as changes in aldosterone, corticosterone, CGRP or ET‐1 levels, might act as either facilitators or compensatory regulators of blood pressure (Ghadhanfar et al., 2014; Neri et al., 2006). Notably, Cargnelli et al. (2000) reported altered vascular reactivity to both α‐adrenergic and ET stimuli following ouabain treatment, despite no significant changes in tail‐cuff blood pressure. Some studies have demonstrated that ouabain‐induced hypertension is potentiated under high‐salt conditions or when co‐administered with deoxycorticosterone acetate (DOCA), suggesting a combinatory effect (Sekihara et al., 1992). Yasujima et al. (1986) and Shigetomi et al. (1989) reported that ouabain normalized noradrenaline‐induced hypertension, emphasizing that ouabain's role may be contextually antihypertensive under certain neurohumoral conditions. These findings support the notion that ouabain may induce subclinical vascular adaptations that do not always translate into overt hypertension.

Measurement methodology should be considered. Although tail‐cuff plethysmography is widely used, it lacks the sensitivity of direct arterial catheterization and may fail to detect subtle or transient changes in blood pressure. However, studies such as those by Li et al. (1995), Wang et al. (1999) and Xavier et al. (2009) reported no significant change in systolic blood pressure after prolonged ouabain treatment, even when invasive techniques were employed. Nevertheless, several other studies have utilized both methods and consistently demonstrated a hypertensive effect (Table 1), suggesting that measurement methodology has a negligible impact on the outcome.

The variability in outcomes also raises the possibility of stress‐induced artifacts. For instance, Manunta et al. (1994) observed that blood pressure increased over time in sham‐treated rats, likely due to procedural stress, which may mask or exaggerate pharmacological effects, as suggested by Li et al. (1995). Furthermore, differences in experimental designs – including ouabain purity, vehicle, frequency of administration and animal handling – may underlie discrepancies across studies.

In some studies, potential explanations are proposed for the lack of a hypertensive response to ouabain treatment (Table 5). Typically, the treatment duration may have been insufficient to induce a significant elevation in blood pressure (e.g. 3 days to 2 weeks; Aileru et al., 2001; Nelissen‐Vrancken et al., 1997; Yasujima et al., 1986). Additionally, the administered ouabain dose was excessively high (e.g., 14.4 mg/kg/day for 2 weeks; Nelissen‐Vrancken et al., 1997), which may paradoxically blunt the hypertensive effect.

**TABLE 5:** Summary of studies demonstrating no hypertensive effects of exogenous ouabain in rats, but a putative explanation is considered.

Strain, sex	Age, weight	BP reading method	Dose	Duration	Route of ouabain administration	BP effect/Comments	References
SD, male	150–250 g	Tail cuff	1.2 mg/kg/day	6 days	i.v. osmotic pump	Antihypertensive effect against noradrenaline infusion	Yasujima et al. (1986)
Wistar, male	260–325 g	Arterial catheter	14.4 mg/kg/day	2 weeks	s.c. osmotic pump	No change	Nelissen‐Vrancken et al. (1997)
		Arterial catheter	14.4 mg/kg/day	2 weeks	s.c. injection	No change (only in rats with MI)	
SD, male	200–250 g	Tail cuff	30 µg/kg/day	3–5 days	s.c. pellet		Aileru et al. (2001)
SD, male	180–220 g	Tail cuff	34 µg/kg loading dose + 27.8 µg/kg/day	4 weeks	i.p. bolus	The group usually observed hypertension after 4 weeks	Jiang, Guo, Lü, Ren et al. (2006)
WKY, male	6 weeks old	Arterial catheter	8 µg/day	5 weeks	s.c. pellet	Controls seem to have high SBP	Xavier et al. (2009)

*n* ranged from 6 to 13 (controls and ouabain‐treated rats). The symbols in the references represent publications from the same or affiliated research groups. BP, blood pressure; i.p., intraperitoneal; MI, myocardial infarction; SBP, systolic BP; s.c., subcutaneous; SD, Sprague–Dawley; WKY, Wistar Kyoto.

Taken together, these findings suggest that ouabain‐induced hypertension is not an absolute effect but rather a conditional outcome that depends on multiple converging factors. As such, researchers aiming to utilize ouabain in experimental hypertension models must carefully consider strain selection, baseline autonomic tone, salt balance, hormonal status and methodological variables. Future studies employing standardized protocols and continuous blood pressure monitoring will be essential to clarify the mechanisms and contexts in which ouabain acts as a hypertensive agent.

## 2 CONCLUSION AND TRANSLATIONAL PERSPECTIVES

Despite significant advances in understanding ouabain‐induced hypertension, several knowledge gaps remain that warrant further investigation. These are summarized in Table 6.

**TABLE 6:** Knowledge gaps and future research directions in ouabain‐induced hypertension.

	Current knowledge	Knowledge gaps	Future directions
Vascular mechanisms	NKA α2 inhibition increases [Ca^2^⁺]i, vasoconstriction, oxidative stress, and vascular remodelling	Differential role of NKA isoforms in various vascular beds Long‐term consequences of remodelling	Develop isoform‐selective NKA modulators Explore vascular bed‐specific effects Study the long‐term impact on vascular structure and function
Renal mechanisms	Modulation of proximal tubular Na^+^ reabsorption and sympathetic regulation	Exact interaction between renal and sympathetic pathways Role of distal nephron segments	Investigate renal‐sympathetic cross‐talk Explore the distal nephron contribution Biomarkers for renal EO‐sensitivity
CNS	OUA acts on MnPO, RVLM, PVN; activates central RAS; enhances sympathetic tone	Relative contribution of CNS vs peripheral EO Long‐term CNS adaptations Role of glial cells and neuroinflammation	Brain imaging studies Target central RAS modulators Investigate neuroimmune pathways
Interindividual variability	OUA's hypertensive effect is strain‐ and condition‐dependent	Genetic determinants of sensitivity Influence of sex hormones, age, and metabolic status	Develop genotype/phenotype‐specific models Study sex‐ and age‐specific mechanisms Personalized risk stratification
Clinical translation	Elevated EO in salt‐sensitive, low‐renin, neurogenic hypertension subgroups	Lack of standardized EO measurement in clinical practice Limited clinical trials on EO‐targeted therapies	Develop EO assays for clinical use Conduct trials with EO‐neutralizing agents and NKA modulators Integrate EO biomarkers into personalized hypertension management

CNS, central nervous system; EO, endogenous ouabain; MnPO, median preoptic nucleus; NKA, Na^+^/K^+^‐ATPase; OUA, ouabain; PVN, paraventricular nucleus; RAS, renin–angiotensin system; RVLM, rostral ventrolateral medulla.

Ouabain‐induced hypertension emerges as a complex and condition‐dependent phenomenon involving intricate interactions across vascular, renal and CNS pathways. At the vascular level, ouabain inhibits NKA, particularly the α2‐isoform, leading to increased [Ca^2^⁺]_i_, augmented vasoconstriction and structural remodelling of resistance arteries. These changes are associated with heightened oxidative stress, inflammation and upregulation of Ca^2^⁺‐mobilizing proteins, collectively contributing to increased peripheral resistance and elevated blood pressure. Renal mechanisms also play a crucial role, with ouabain modulating tubular sodium handling – especially within the proximal nephron – and engaging sympathetic neural circuits, thereby promoting Na^+^ retention and volume expansion.

CNS mechanisms are particularly salient in ouabain‐induced hypertension. Ouabain influences Na^+^‐sensitive brain regions such as the MnPO, RVLM and PVN, where it enhances sympathetic outflow and impairs baroreflex function. These effects are tightly linked to the central RAS and are often independent of peripheral ouabain levels, highlighting the significance of brain‐derived EO‐like compounds in neurogenic forms of hypertension.

Importantly, the hypertensive effect of ouabain is not universally observed. Certain variability exists among different rodent strains and experimental protocols. Genetic predisposition, baseline autonomic tone, hormonal status, salt balance and methodological differences might influence the development and severity of the hypertensive phenotype. This variability emphasizes that ouabain functions not as a universal hypertensinogen but as a modulator whose pressor effect depends on the convergence of permissive physiological and experimental conditions.

Although the mechanistic understanding of ouabain‐induced hypertension has largely emerged from preclinical models, growing evidence supports a significant role for EO in the pathophysiology of human hypertension. EO has been identified in human plasma and tissues using advanced techniques, including liquid chromatography and mass spectrometry, confirming its structural identity with the plant‐derived compound (Hamlyn et al., 1991; Manunta et al., 2001). Elevated plasma levels of EO have been observed in patients with essential hypertension (Manunta et al., 2000), predominantly in a subset of those with salt‐sensitive or resistant hypertension (Doris & Bagrov, 1998; Manunta, Hamilton, Hamlyn, 2006), primary aldosteronism (Ferrandi et al., 2004), chronic kidney disease (Ferrandi et al., 2005) and preeclampsia (Fedorova et al., 2005), suggesting a broad relevance of ouabain across Na^+^‐retentive and vasoconstrictive pathologies as well as hinting at synergistic interactions between EO and other pressor systems.

In humans, the NKA α2 isoform, which is highly expressed in vascular smooth muscle cells, shows particular sensitivity to ouabain (Blaustein et al., 2016). Genetic studies have revealed that polymorphisms in *ATP1A1* and *ATP1A3* – genes encoding α1 and α3 subunits – may influence susceptibility to hypertension by altering ouabain binding or signal transduction efficacy (Wang et al., 2014). Such findings underscore the potential for isoform‐selective ouabain sensitivity to contribute to interindividual variability in blood pressure regulation.

Experimental data also demonstrate translational parallels in renal Na^+^ handling. Ouabain has been shown to downregulate dopamine D1 receptor signalling in human renal proximal tubules, which may lead to impaired natriuresis and Na^+^ retention (Armando et al., 2015; Zeng et al., 2008). This mechanism may underlie the heightened salt sensitivity observed in subgroups of hypertensive patients. Moreover, clinical studies have identified increased sympathetic nervous system activity and reduced baroreflex sensitivity in essential hypertension, aligning with central effects of ouabain observed in rodent models.

The CNS component of ouabain's hypertensinogenic action is increasingly recognized in humans. CVOs, such as the SFO and OVLT, lack a complete blood–brain barrier and are sensitive to changes in cerebrospinal fluid Na^+^ levels. These brain regions are capable of responding to Na^+^ and ouabain to regulate sympathetic tone and blood pressure (Blaustein & Hamlyn, 2020). In hypertensive patients, altered Na^+^ sensing and potential EO release in the CNS may contribute to sympathetic overactivity and sustained hypertension.

Clinically, the importance of ouabain extends beyond its pathophysiological role. Plasma EO levels have been proposed as biomarkers for hypertension risk and treatment responsiveness (Manunta et al., 2009). Therapeutically, rostafuroxin, a digitoxigenin derivative designed to disrupt EO–NKA interactions, reduced blood pressure in EO‐hypertensive rats and showed potential in early‐phase human trials (Lanzani et al., 2010), though larger clinical validation is pending (Manunta et al., 2016). Thus far, efforts are underway to develop selective NKA signalling antagonists – compounds that inhibit ouabain‐mediated signal transduction without impairing the pump's ion transport function. Such agents hold promise for targeting EO‐related hypertensive states, especially in patients with salt sensitivity or resistance to conventional therapy.

Personalized approaches to hypertension may benefit from identifying biomarkers of EO sensitivity and refining phenotypic characterization of patients based on central and peripheral Na^+^ handling. Additionally, the development of strain‐ or genotype‐specific preclinical models will be critical for bridging experimental findings with human pathophysiology. Understanding the dual roles of ouabain as a compensatory modulator under physiological conditions and as a hypertensive trigger under specific pathological states may inform the design of novel therapeutic paradigms, particularly in resistant or complex forms of hypertension.

## AUTHOR CONTRIBUTIONS

Both authors contributed to the conception of the review and contributed to the design, draft, and final version of the manuscript, as well as the critical revision. Both authors have approved the final version of the manuscript and agree to be accountable for all aspects of the work in ensuring that questions related to the accuracy or integrity of any part of the work are appropriately investigated and resolved. All persons designated as authors qualify for authorship, and those who qualify for authorship are listed.

## CONFLICT OF INTEREST

None declared.
